# Sufentanil sublingual tablet system for enhanced recovery after total knee arthroplasty: a prospective observational case study

**DOI:** 10.1186/s13741-022-00284-x

**Published:** 2022-10-04

**Authors:** Emmanuel Rineau, Benjamin Dumartinet, Emmanuel Samson, Apolline Dollfus, Corentin Aubourg, Sigismond Lasocki

**Affiliations:** 1 Department of Anesthesiology and Intensive Care, University Hospital and Health Faculty of the University of Angers, 4 rue Larrey, 49100 Angers, France; 2 Department of Anesthesiology and Intensive Care, University Hospital of Rennes, Rennes, France

**Keywords:** Postoperative recovery, Postoperative pain, Prosthetic knee surgery, Sufentanil sublingual tablet systems, Patient-controlled analgesia

## Abstract

**Background:**

Postoperative pain is one of the main factors that delays recovery after prosthetic knee surgery. The use of sufentanil sublingual tablet systems (SSTS) can effectively relieve postoperative pain, but their value in facilitating early mobilization has been little studied so far. Our aim here was to assess whether their use could facilitate recovery after knee arthroplasty in an enhanced recovery program.

**Case presentation:**

In a prospective observational single-center study, thirty patients operated on for primary knee arthroplasty in the enhanced recovery pathway were included. Patients who received the SSTS (*n*=15) were compared with those who received an intravenous morphine patient-controlled analgesia (PCA) (*n*=15). Our recovery program included in particular the use of an adductor canal block, periarticular infiltration of local anesthetic by the surgeon, removal of the venous cannula from the recovery room if possible, the use of an SSTS when available or an IV morphine PCA otherwise, and early physiotherapy. Recovery parameters including the Timed-Up and Go test, pain scores at rest and on exertion, knee flexions, complications, and lengths of hospital stay were not significantly different between the two groups. However, the postoperative opioid consumption in morphine equivalents was significantly greater in the SL-sufentanil group and these patients had their venous cannula removed earlier than in IV-morphine group.

**Conclusion:**

In our center, the use of a SSTS was suitable for treating postoperative pain after knee arthroplasty, but it did not improve early recovery in comparison with a morphine PCA.

**Supplementary Information:**

The online version contains supplementary material available at 10.1186/s13741-022-00284-x.

## Background

Enhanced recovery after surgery programs has shown their interest in reducing both complications and length of hospital stay, especially in prosthetic knee surgery (Wainwright et al. 2020). One of the imperatives of these programs is to control postoperative pain, since it may impair patient’s mobilization and lengthen hospitalization (Soffin and YaDeau 2016; Wainwright et al. 2020).

As in other surgeries, multimodal analgesia is recommended for prosthetic knee surgery and frequently includes a regional analgesia technique. Nowadays, the femoral nerve block has been gradually replaced by periarticular infiltrations or more distal blocks, such as the adductor canal block, in order to facilitate postoperative mobilization (Wainwright et al. 2020). However, although these techniques avoid the motor block of the quadriceps, they often provide less coverage of the painful operating area compared to the femoral nerve block (Kuang et al. 2017). Opioid treatments are therefore still frequently prescribed and, as a result, require either the maintenance of an intravenous line (for opioid infusion using patient-controlled analgesia (PCA)) or the use of oral opioids given by ward nurses (Lamplot et al. 2014).

The sufentanil sublingual tablet system (SSTS, Zalviso®) is a recent therapeutic alternative that can be used for acute postoperative pain (van de Donk et al. 2018). It has shown its interest in reducing postoperative pain in various surgeries, including prosthetic knee surgery (Jove et al. 2015). Due to its short onset of action (about 6 min) permitted by the sublingual route and the non-requirement of a venous line for its administration, this device could be of interest in facilitating rapid mobilization, and therefore, early rehabilitation after this type of functional surgery, compared to intravenous or oral opioids usually prescribed in the postoperative period. However, to our knowledge, its value in facilitating early mobilization after this surgery has not been precisely evaluated yet.

In the orthopedic unit of our hospital, we have implemented early recovery programs, depending on the type of surgery concerned. For knee prosthetic surgery, these programs include in particular the use of an adductor canal block (instead of the use of a perinervous femoral catheter), periarticular infiltration of local anesthetic by the surgeon, removal of the venous cannula from the recovery room if possible (instead of between day 1 to day 3 after surgery), the use of an SSTS (instead of an IV morphine PCA), and early physiotherapy (from the day of the surgery if possible). However, due to the presence in our unit of three SSTS devices only, patients for whom the SSTS device is not available receive an IV morphine PCA, even when they are included in the early recovery pathway. In this study, our aim was to assess whether the use of SSTS could have facilitated early recovery after total knee arthroplasty, in comparison with intravenous morphine PCA followed by oral oxycodone, by using objective criteria of postoperative physiotherapy.

## Case presentation

### Design and patients

In order to assess the effectiveness of our enhanced recovery program, and the potential interest of using SSTS in our unit, we conducted a prospective observational single-center study at Angers University Hospital between March 1, 2018, and March 1, 2019. The study was approved by the local Ethical Committee (Comité d’Ethique du CHU d’Angers, reference number 2018-13). Inclusion criteria were patients scheduled to be operated on for primary unilateral knee arthroplasty in the enhanced recovery pathway of our center and age ≥ 18 years old.

Two groups of patients were compared in this case study, those who received the SSTS (SL-sufentanil group) and those who received a morphine PCA (IV-morphine group). To assess early recovery, the primary endpoint of our study was the postoperative TUG measured at day 1, day 2, and day 3 after surgery. Secondary endpoints included perioperative pain (assessed by the numerical rating scale of pain), opioid consumption, opioid adverse effects, knee flexion, time of the venous cannula removal, length of stay in the postanesthesia care unit (PACU), and total length of hospital stay. Morphine equivalents were calculated according to one of the usual rules (Shaheen et al. 2009) which was here: oral oxycodone 1 mg = intravenous morphine 0.5 mg and sublingual sufentanil 15 μg = intravenous morphine 2.5 mg.

Results are presented as numbers (%) and medians [interquartiles 25–75%]. Categorical variables were compared between groups using Fisher’s test. Qualitative variables were compared between groups or between perioperative times (for the TUG) using Mann-Whitney test. Statistical analyses were performed using the JMP software (SAS, USA).

### Perioperative management of patients in the enhanced recovery pathway

Total knee arthroplasties were performed under general or spinal anesthesia, and unless contraindicated, all patients received both a periarticular infiltration and a preoperative adductor canal block.

The SSTS was allocated as usual in our center, according to its availability (3 devices only in our unit) when the patient arrived in the recovery room after surgery, and was started from the recovery room for a maximal period of 72 h. Patients for whom a SSTS device was not available were prescribed an intravenous morphine PCA (settings: bolus 1 mg, refractory period: 7 min, maximum dose: 20 mg for 4 h) for a maximum duration of 72 h. The computerized prescription of our unit procedure indicated however to relay the morphine PCA as soon as possible by oral immediate release oxycodone (5 mg on demand every 4 h for pain with numerical scale above than 3) and to remove the peripheral venous cannula as soon as an intravenous infusion was no longer needed. In PACU, analgesic titration was performed with intravenous morphine for patients in the IV-morphine group, although it could be performed immediately with the STSS and/or with IV morphine in the SL-sufentanil group.

In the enhanced recovery pathway of our postoperative orthopedic unit, all patients received early postoperative physiotherapy, including a daily Timed Up and Go Test (TUG) when possible. This test has been well evaluated and validated to measure physical recovery after surgery (Alrawashdeh et al. 2021). Knee flexion measurements were recorded by the physiotherapists as usual. No change was made in the perioperative management of patients.

### Preoperative characteristics and intraoperative data

Thirty patients were included between March 2018 and March 2019, 15 in the IV-morphine group and 15 in the SL-sufentanil group. The pre- and intra-operative characteristics of the patients were similar between the 2 groups, in particular concerning the preoperative analgesic consumption, the duration of the procedure, and the anesthetic techniques used (Tables 1 and 2).

**Table 1:** Preoperative data

	IV-morphine group (*n* = 15)	SL-sufentanil group (*n* = 15)	*p*
Characteristics			
Age (years)	69 [62-76]	75 [68-78]	0.16
Female sex	11 (73%)	6 (40%)	0.13
BMI (kg/m^2^)	27 [25-32]	29 [26-32]	0.75
Diabetes	5 (33%)	5 (33%)	1
HTA	8 (53%)	8 (53%)	1
Active smoking	1 (6%)	1 (6%)	1
Alcohol intake	0 (0%)	0 (0%)	-
Anxio-depressive disorder	3 (20%)	0 (0%)	0.22
Analgesic medications			
Paracetamol	8 (53%)	7 (46%)	1
Tramadol	0 (0%)	2 (13%)	0.48
NSAID	1 (6%)	2 (13%)	1
Codein	1 (6%)	0 (0%)	1
Anticonvulsivant drug	1 (6%)	1 (6%)	1
Antidepressant drug	2 (13%)	2 (13%)	1
Knee condition			
Knee flexion (degrees)	122 [108-145]	117 [110-123]	0.47
Pain at rest (NRS)	1 [0-5]	2 [0-5]	0.82

Values are medians [interquartiles 25–75%] or numbers (%)

*BMI* body mass index, *NSAID* nonsteroidal anti-inflammatory drug, *NRS* numerical rating scale of pain

**Table 2:** Intra-operative data

	IV-morphine group (*n* = 15)	SL-sufentanil group (*n* = 15)	*p*
Surgical data			
Operated side	3 (20%)	7 (46%)	0.24
Use of a tourniquet	12 (80%)	14 (93%)	0.59
Surgical infiltration	14 (93%)	14 (93%)	1
Ropivacain dose for infiltration (mg)	235 [200–300]	245 [200–300]	0.98
Duration of surgery (min)	90 [83–106]	107 [88–122]	0.11
Anesthetic data			
General anesthesia	14 (93%)	12 (80%)	0.59
Use of IV sufentanil (vs remifentanil)	11 (73%)	12 (80%)	1
Total IV sufentanil dose (μg)	35 [30–50]	37.5 [31.3–47.5]	0.72
Spinal anesthesia	1 (6%)	3 (20%)	0.59
Intrathecal bupivacain dose (mg)	15 [15–15]	15 [12.5–15]	-
Intrathecal sufentanil dose (μg)	5 [5–5]	5 [5–5]	-
Adductor canal block	14 (93%)	14 (93%)	1
Ropivacain dose used for block (mg)	40 [20–95]	40 [37.5–90]	0.79
Others analgesics			
IV dexamethasone	14 (93%)	9 (60%)	0.08
Dexamethasone dose (mg)	2 [0–5]	2 [0–4]	0.65
IV ketamine	12 (80%)	7 (47%)	0.13
Total ketamine dose (mg)	20 [15–23.7]	20 [15–25]	0.69
IV paracetamol	15 (100%)	14 (93%)	1
Paracetamol dose (g)	1 [1–1]	1 [1–1]	1
IV ketoprofene	12 (80%)	8 (53%)	0.25
Ketoprofene dose (mg)	100 [100–100]	100 [100–100]	0.26
IV nefopam	8 (53%)	9 (60%)	1
Nefopam dose (mg)	20 [20–20]	20 [20–20]	1

Values are medians [interquartiles 25–75%] or numbers (%)

*IV* intravenous

### Postoperative physical rehabilitation

**Fig. 1:**
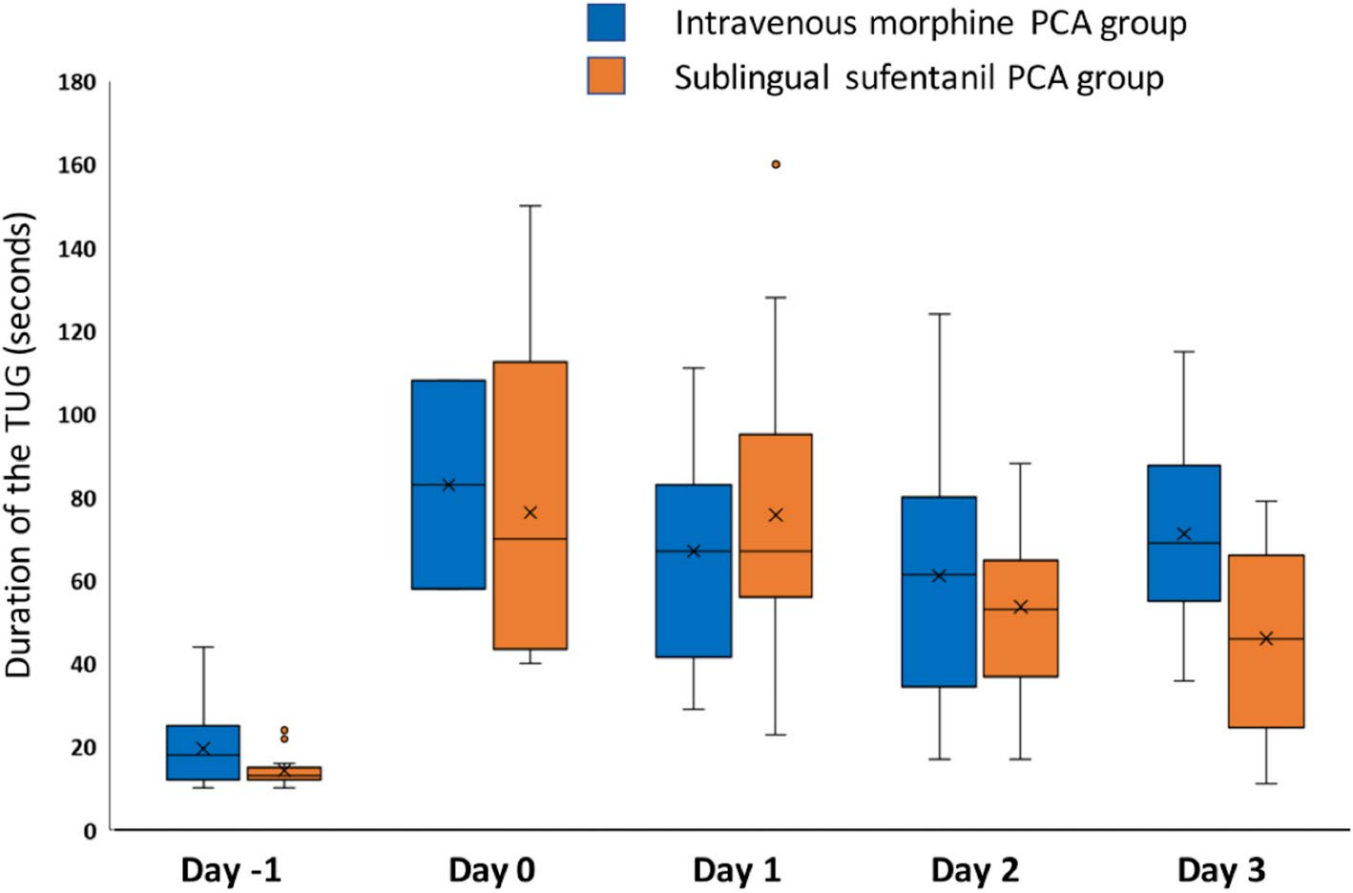
Perioperative times obtained with the Timed Up and Go (TUG) test. There were no significant differences between the IV-morphine and SL-sufentanil groups from day 1 to day 3

There were no significant differences in terms of postoperative physical rehabilitation assessed by the TUG between the two groups from day 1 to day 3 after surgery (Fig. 1), nor between the postoperative/preoperative TUG ratios at these different times. In addition, knee mobilities were similar between the 2 groups (Table 3).

**Table 3:** Postoperative data related to pain and mobilization

	IV-morphine group (*n* = 15)	SL-sufentanil group (*n* = 15)	*p*
Total of IV morphine equivalents consumed (PACU and ward)	13 [8.5–19]	40 [9.5–70]	0.03
Morphine equivalents consumed in the PACU	6 [0–8]	2.5 [2.5–5]	0.93
Morphine equivalents consumed in the ward (mg)	8 [3.8–13]	37.5 [11.3–58.8]	0.03
Opioid consumption in the PACU			
IV morphine or SSTS titration	12 (80%)	15 (100%)	0.22
IV morphine titration	12 (75%)	4 (27%)	<0.01
IV morphine dose (when received) (mg)	6.5 [5.5–8.5]	6 [3–7.5]	0.47
SSTS titration	-	13 (100%)	-
Number of requested bolus	-	1 [1–2]	-
Number of received bolus	-	1 [1–2]	-
Sublingual sufentanil (μg)	-	15 [15–30]	-
Opioid consumption in the surgical ward			
Total of requested bolus on PCA or SSTS	3 [2–9]	15 [4.5–25]	0.11
Total of received bolus on PCA or SSTS	3 [2–10.5]	15 [4.5–23]	0.09
Total of oxycodone tablets consumed	1 [0.5–2]	0 [0–0]	<0.01
Total of IV morphine equivalents consumed (mg)	8 [3.8–13]	37.5 [11.3–58.8]	0.03
Total of IV morphine (mg)	3 [2–10.5]	-	-
Total of sublingual sufentanil (μg)	-	225 [67.5–345]	-
Total of oral oxycodone (mg)	5 [2.5–10]	0 [0–0]	<0.01
Postoperative pain at rest			
NRS day 0	1.5 [1–2]	2 [1–3]	0.61
NRS day 1	2 [1.6–3]	1.6 [1–2.6]	0.19
NRS day 2	1 [0–2]	1.6 [0.3–3.7]	0.27
NRS day 3	1.3 [0.5–2.2]	1.5 [0.3–2]	0.88
Postoperative pain on exertion			
NRS day 0	3 [2–6]	4 [1–6]	0.44
NRS day 1	4 [3.8–5.3]	6 [4–7]	0.12
NRS day 2	4 [2–6]	4 [3–6]	0.91
NRS day 3	4 [2–6]	2 [1.3–4.8]	0.19
Opioid-related side effects			
Nausea and vomiting	4 (27%)	6 (40%)	0.70
Constipation	2 (13%)	2 (13%)	1
Peripheral venous cannula withdrawn			
Day 0	0 (0%)	12 (80%)	<0.01
Day 1	0 (0%)	15 (100%)	<0.01
Day 2	7 (47%)	15 (100%)	<0.01
Day 3	13 (87%)	14 (93%)	0.55
Flexion of the operated knee (degrees)			
Day 0	80 [80–80]	82 [78–86]	0.59
Day 1	80 [60–90]	80 [60–90]	0.80
Day 2	90 [80–95]	90 [80–90]	0.65
Day 3	90 [87–90]	90 [86–90]	1
Length of stay in postanesthesia care unit (min)	137 [125–164]	133 [115–145]	0.48
Length of hospital stay (days)	4 [4–6]	5 [4.5–7]	0.34

Values are medians [interquartiles 25–75%] or numbers (%)

*IV* intravenous, *NRS* numerical rating scale of pain, *PACU* postanesthesia care unit, *PCA* patient-controlled analgesia system, *SL* sublingual, *SSTS* sufentanil sublingual tablet system

### Postoperative pain

Postoperative pain measured at rest and on exertion (during the TUG) was not significantly different between the two groups from day 0 to day 3 (Table 3). The total of IV morphine equivalents consumed in the surgical ward or both in the PACU and the surgical ward was significantly greater in the SL-sufentanil group. The number of requests and really received doses with either the SSTS or the morphine-PCA were also greater, although not significantly, in the SL-sufentanil group. However, the number of oxycodone tablets used was significantly greater in the IV-morphine group. The number of side effects attributable to opioid use was similar between the IV-morphine and SL-sufentanil groups (Table 3).

### Other results

Patients in the SL-sufentanil group had their peripheral venous cannula removed earlier than in IV-morphine group, but there were no significant differences in the length of stay in the postanesthesia care unit and in the total length of hospital stay (Table 3).

Finally, as outcomes regarding postoperative pain and mobilization may differ depending of the type of anesthesia received (Bourget-Murray et al. 2022; Johnson et al. 2016; Memtsoudis et al. 2019; Sciberras et al. 2022), we conducted a post hoc analysis in patients operated on under general anesthesia only (i.e., after removal of patients who have had a spinal anesthesia). Results of comparisons between the IV-morphine group and the SL-sufentanil group were similar than those obtained on the whole group patients, with in particular a greater morphine equivalents consumption in the SL-sufentanil group and no significant differences in postoperative pain, times to complete the TUG, knee flexions, and lengths of hospital stay (Additional file 1, Table S1). Only pain on exertion at day 1 was significantly higher in the SL group (Table S1).

## Discussion

In the present study, the use of a sublingual sufentanil PCA for patients included in the enhanced recovery program did not improve postoperative rehabilitation after knee surgery in comparison with IV morphine PCA, either in terms of functional capacities assessed by the TUG, nor in terms of knee flexion or length of hospital stay.

Early rehabilitation is an important goal after prosthetic knee surgery as it can improve postoperative functional outcomes and limit perioperative complications (Zhu et al. 2017). However, several aspects of rehabilitation can be measured in the postoperative period (Alrawashdeh et al. 2021). We have chosen here to measure the TUG because it is an easy-to-perform validated and objective parameter involving both joint mobility, muscle strength, and the patient’s fatigue (Podsiadlo and Richardson 1991; Yeung et al. 2008). In our study, the use of this parameter as the primary endpoint did not allow us to observe any significant differences between the 2 groups. While the low number of patients may partly explain the lack of difference, no trend was visible between the two groups on other recovery parameters, such as knee flexion, pain, and length of hospitalization. These data confirm the limited interest of SSTS in accelerating rehabilitation, already observed in a very recent study which evaluated its interest in reducing pain and time to first mobilization (Noel et al. 2020).

Pain appeared however to be effectively controlled in both groups, confirming the efficacy of SSTS for the control of postoperative pain after knee arthroplasty (Jove et al. 2015; Meijer et al. 2018; Melson et al. 2014; Scardino et al. 2018). But, although its use has been shown to be effective in treating postoperative pain in various surgeries, including major orthopedic surgery, no clinically significant difference has been observed in comparison with other usual effective analgesic techniques such as the PCA morphine so far (Noel et al. 2020; van Veen et al. 2018). Conversely, it has even been shown to be responsible for more side effects, in particular nausea and vomiting (Jove et al. 2015; Noel et al. 2020; van Veen et al. 2018). Even if we did not observe significantly more adverse effects of morphine, we observed in our case study a significantly higher consumption in terms of morphine equivalents.

One limit of SSTS is probably the short duration of effect of sublingual sufentanil, in comparison with intravenous morphine and oral morphine or oxycodone. It probably explains the larger number of requests that were done using the SSTS, compared with IV morphine PCA. The benefit of self-controlled oral analgesia, including opioids if necessary, probably needs to be further evaluated, especially in the context of accelerated rehabilitation.

Interestingly, the use of SSTS allowed us to withdraw the peripheral venous cannula earlier than in the morphine PCA group. Thus, also we confirmed here that the early removal of the venous cannula is feasible, even after prosthetic orthopedic surgery, we did not confirm our primary hypothesis that the absence of IV line connected to a PCA device would improve mobilization and earlier discharge from hospital.

In conclusion, while the use of SSTS appears to be suitable for treating postoperative pain after knee arthroplasty, additional data obtained from randomized controlled trials, but now focusing on mobilization or specific postoperative recovery score (Léger et al. 2021; Yuksel et al. 2017), are needed to assess its benefit for enhanced recovery and to evaluate its medico-economic potential benefit.

## Supplementary Information

**Additional file 1: Table S1.** Postoperative data related to pain and mobilization in patients operated on under general anesthesia.

## Data Availability

The datasets used and/or analyzed during the current study are available from the corresponding author on reasonable request.

## Abbreviations

IV: Intravenous
PCA: Patient-controlled analgesia
SL: Sublingual
SSTS: Sufentanil sublingual tablet system
TUG: Timed Up and Go Test
