# Supplementary material for: Sufentanil sublingual tablet system for enhanced recovery after total knee arthroplasty: a prospective observational case study
Source: Perioper Med (Lond). 2022 Oct 4;11:50. doi: 10.1186/s13741-022-00284-x (PMC9531461; doi:10.1186/s13741-022-00284-x)
Supplement: Supplementary file 1 — Additional file 1: Table S1. Postoperative data related to pain and mobilization in patients operated on under general anesthesia. [file 13741_2022_284_MOESM1_ESM.docx]

**Supplementary material**

**Sufentanil sublingual tablet system for enhanced recovery after total knee arthroplasty: a prospective observational case study**

Emmanuel Rineau ^1*^, Benjamin Dumartinet^1^, Emmanuel Samson^1^, Apolline Dollfus^2^, Corentin Aubourg^1^, Sigismond Lasocki^1^

^1^ Department of Anesthesiology and Intensive Care, University Hospital and Health Faculty of the University of Angers, Angers, France.

^2^ Department of Anesthesiology and Intensive Care, University Hospital of Rennes, Rennes, France.

*Correspondance to Emmanuel Rineau:

Address : Département Anesthésie Réanimation, CHU Angers, 4 rue Larrey, 49100 Angers, France

Telephone number: +33 241353635

E-mail: [erineau@live.fr](mailto:erineau@live.fr)

| **Table S1. Postoperative data related to pain and mobilization in patients operated on under general anesthesia** | | | |
| --- | --- | --- | --- |
|  | IV-morphine group (n = 14) | SL-sufentanil group (n = 12) | p |
| **Pain and opioid consumption** | | | |
| Total of IV morphine equivalents consumed (PACU and ward) | 13.5 [7.6-20.5] | 34.3 [12.1-75.6] | 0.04 |
| Morphine equivalents consumed in the PACU | 5 [0-7.3] | 3.5 [2.5-5.8] | 0.77 |
| Morphine equivalents consumed in the ward (mg) | 8.3 [4.1-16] | 31.3 [8.1-72.5] | 0.04 |
| Opioid consumption in the PACU |  |  |  |
| IV morphine or SSTS titration | 11 (79%) | 12 (100%) | 0.22 |
| IV morphine titration | 11 (79%) | 4 (33%) | 0.04 |
| Opioid consumption in the surgical ward |  |  |  |
| Total of requested bolus on PCA or SSTS | 4.5 [1.5-10.8] | 12.5 [3.3-35.3] | 0.15 |
| Total of received bolus on PCA or SSTS | 4.5 [1.5-13.5] | 12.5 [3.3-28.8] | 0.13 |
| Total of IV morphine (mg) | 4.5 [2-11.8] | - | - |
| Total of sublingual sufentanil (µg) | - | 187.5 [86.3-333.8] | - |
| Total of oral oxycodone (mg) | 7.5 [3.8-12.5] | 0 [0-0] | <0.01 |
| Postoperative pain at rest |  |  |  |
| NRS Day 0 | 1.5 [1-2.1] | 2 [1-3] | 0.57 |
| NRS Day 1 | 2.2 [1.9-3.2] | 1.6 [1-2.5] | 0.12 |
| NRS Day 2 | 1 [0-2] | 1.8 [0.4-.3.8] | 0.28 |
| NRS Day 3 | 1.3 [0.5-2.2] | 1 [0.3-2] | 0.78 |
| Postoperative pain on exertion |  |  |  |
| NRS Day 0 | 8 [8-8] | 4 [1-7] | 0.37 |
| NRS Day 1 | 4 [4-5.5] | 6 [5-7.8] | 0.04 |
| NRS Day 2 | 4 [2-6] | 4.5 [2.3-6] | 0.98 |
| NRS Day 3 | 4 [2-6] | 2 [1.5-4.5] | 0.24 |
| Opioid-related side effects |  |  |  |
| Nausea and vomiting | 4 (29%) | 5 (42%) | 0.68 |
| Constipation | 2 (14%) | 2 (17%) | 1 |
| **Mobilization and length of hospital stay** | | | |
| Timed Up and Go test, time to complete (seconds) |  |  |  |
| TUG Day -1 | 17 [11.8-22] | 13 [10.5-15] | 0.16 |
| TUG Day 0 | 58 [58-58] | 75 [57.5-112.5] | - |
| TUG Day 1 | 65 [39-84] | 79 [57-113.8] | 0.28 |
| TUG Day 2 | 66 [32-84] | 56.5 [40.5-80] | 0.64 |
| TUG Day 3 | 62 [46-85.5] | 58.7 [35.5-70] | 0.29 |
| Flexion of the operated knee (degrees) |  |  |  |
| Flexion Day 0 | 80 [80-80] | 82.5 [76.2-85] | 1 |
| Flexion Day 1 | 80 [60-90] | 80 [60-85] | 0.86 |
| Flexion Day 2 | 90 [80-95] | 87.5 [80-90] | 0.27 |
| Flexion Day 3 | 90 [87.5-90] | 90 [82.5-90] | 0.43 |
| Length of stay in postanesthesia care unit (min) | 133.5 [125-156.3] | 134.5 [117.5-145] | 0.62 |
| Lenght of hospital stay (days) | 4.5 [4-6.5] | 5.5 [4.3-7.8] | 0.41 |
| Values are medians [interquartiles 25% -75%] or numbers (%) of non-missing values.  IV, intravenous; NRS, numerical rating scale of pain; PACU, postanesthesia care unit; PCA, patient-controlled analgesia system; SL, sublingual; SSTS, sufentanil sublingual tablet system; TUG, Timed Up and Go test. | | | |
